# Neuroenhancement and neuroprotection by oral solution citicoline in non-arteritic ischemic optic neuropathy as a model of neurodegeneration: A randomized pilot study

**DOI:** 10.1371/journal.pone.0220435

**Published:** 2019-07-26

**Authors:** Vincenzo Parisi, Lucilla Barbano, Antonio Di Renzo, Gianluca Coppola, Lucia Ziccardi

**Affiliations:** 1 IRCCS—Fondazione Bietti, Rome, Italy; 2 Department of Medico-Surgical Sciences and Biotechnologies, Sapienza University of Rome—Polo Pontino, Latina, Italy; Univerity of Brescia, ITALY

## Abstract

**Purpose:**

To evaluate whether treatment with Citicoline in oral solution (OS-Citicoline) would increase visual function, retinal ganglion cells (RGCs) function, and neural conduction along visual pathways (neuroenhancement), and/or induce preservation of RGCs fibers’ loss (neuroprotection) in non-arteritic ischemic optic neuropathy (NAION), a human model of neurodegeneration.

**Methods:**

Thirty-six patients with NAION and 20 age-matched controls were enrolled. Nineteen NAION patients received 500 mg/day of OS-Citicoline for a 6-month period followed by 3-month of wash-out (NC Group); 17 NAION patients were not treated (NN Group) from baseline to 9 months. In all subjects at baseline, and in NC and NN eyes at 6 and 9 months of follow-up, we assessed Visual Acuity (VA), Pattern Electroretinogram (PERG), Visual Evoked Potentials (VEP), retinal nerve fiber layer thickness (RNFL-T), and Humphrey 24–2 visual field mean deviation (HFA MD). Mean differences were statistically evaluated with ANOVA between Groups, and linear correlations were analysed with Pearson’s test.

**Results:**

At 6 months, significant differences between groups for all parameters were observed (ANOVA, p<0.01). In NC eyes, VA increased, PERG responses increased, VEP recordings improved and were significantly correlated with increases in HFA MD (p<0.01), and RNFL-T was unmodified or improved. In contrast, in NN eyes, VA, PERG, VEP responses, RNFL-T, and HFA MD were further worsened. Significant differences were still present at 9-month follow-up in the NN Group and after 3 months of OS-Citicoline wash-out in NC eyes.

**Conclusions:**

OS-Citicoline treatment induced neuroenhancement (improvement in RGCs function and neural conduction along visual pathways related to improvement of visual field defects) and neuroprotection (unmodified or improved RNFL morphological condition) in a human model of NAION involving fast RGCs degeneration.

**Trial registration:**

ClinicalTrials.gov NCT03758118.

## Introduction

Neurodegeneration is a common feature of brain and/or eye diseases characterised by ongoing pathological loss of neuronal structure and function with consequent cell death by apoptosis [1], necrosis [2], or necroptosis. [3,4]

The degeneration of retinal ganglion cells (RGCs) and their fibers which form the optic nerve can be an expression of neurodegenerative phenomena that may occur in the presence of ocular diseases (i.e., glaucoma and ischemic optic neuropathy) or due to mechanisms of primary or retrograde degeneration in pathologies involving the Central Nervous System (i.e., Alzheimer’s disease, Parkinson’s disease, and demyelinating optic neuritis) [5–8].

Non-arteritic anterior ischemic optic neuropathy (NAION) is an irreversible, painless, and acute vascular failure of the optic nerve. NAION is characterised by sudden loss of visual acuity and visual field. [9] Psychophysics progressively worsens with regard to functional and structural neuronal impairment [10], as described by unrelated retinal dysfunction and impairment of neural conduction along visual pathways [11], reduced Retinal Nerve Fiber Layer (RNFL) thickness assessed by optical coherence tomography (OCT) [12], and flow impairment of retinal and choriocapillaris peripapillary capillaries [13,14]. In particular, the loss of RGCs fibers (assessed by RNFL thickness) has been reported in a period of 1 to 6 months from the acute injury. [15,16] Given these abnormal morpho-functional characteristics, Khalilpour et al. [17] have considered NAION a valuable model of neurodegeneration of the retinal structures forming the optic nerve.

A therapeutic approach based on neuroprotection alongside correction of systemic circulatory parameters could prevent progression of visual deficits and rescue neuronal loss. Indeed, in the field of vision, neuroprotection, neurorecovery or neuroenhancement, neurorescue, axoprotection, and neurorepair are potential interventions for maintaining RGCs and morpho-functional integrity of their axons after injury.

Briefly, neuroprotection is the relative preservation of neuronal structure and/or function with mechanisms able to defend the visual nervous system against neuronal injury due to acute or chronic neurodegenerative disorders. [18] Neurorecovery or neuro-enhancement relies on the notion that RGCs are sick but not irreversibly dying, and it consists of the complete or partial short-term restoration of living, non-functioning, or poorly functioning neurons back to functional health. [19] Neurorescue is the combination of neuroprotection and neuro-enhancement. [20] Axoprotection is an approach with the goal of maintaining persistent axonal integrity and function after injury. [21] Neuroregeneration or neurorepair refers to any strategy that can increase survival and axonal growth to partly or entirely rebuild long-standing connections between the eye and brain for visual restoration by replacing lost connections in the visual nervous system [22–24].

To achieve these targets, complimentary primary therapies such as reducing inflammation, lowering IOP, controlling risk factors, improving optic nerve head circulation, and/or administration of many substances have been tested in clinical studies for diseases of the optic nerve, particularly glaucoma [25–27].

Among these, Citicoline (cytidine-5’-diphosphocholine) is an endogenous compound that can be administered exogenously with proven neuroenhancement effects in many brain and ocular diseases characterised by hypoxia and ischemia [28], such as stroke [29,30], brain trauma [31], Parkinson’s disease [32,33], Alzheimer’s disease [29,34,35], glaucoma [36–40], and NAION [41,42].

Experimental studies suggest neuromodulatory effects and protective roles of Citicoline on RGCs [43–45] due to its membrane protective potential [46] and ability to stimulate certain neurotransmitters including dopamine, known to be largely expressed in both the retina and post-retinal visual pathways [47–50].

Since 2005, Citicoline has been available for oral administration. We previously reported improvements in RGCs function and neural conduction along small axons forming the visual pathways after treatment with Citicoline in a pilot study employing a neurodegenerative model of NAION with 1600 mg oral suspension. [41] Citicoline is marketed as a new formulation of oral solution with bio-availability similar to that of endovenous administration. [51,52] Ottobelli et al. observed a slow rate of progression of visual field deficits in glaucoma following treatment with 500 mg/day of Citicoline in oral solution (OS-Citicoline) [53].

Therefore, the purpose of our study was to evaluate whether treatment with OS-Citicoline would increase visual function, RGCs function (evaluated by Pattern Electroretinogram [PERG]) and neural conduction along visual pathways (assessed by Visual Evoked Potentials [VEP]) and/or to induce preservation of RGCs fibers loss (quantified by OCT) in NAION as a human model of neurodegeneration.

Results from the present study would clarify whether OS-Citicoline exclusively induces functional improvement of RGCs and visual pathways, which is well established in glaucoma (neuroenhancement) [19, 36–40], or sparing of RGCs fibers loss (neuroprotection) as suggested by experimental studies (see Parisi et al. (2018) for a review [45]).

In addition, our work aimed to detect whether the possible morpho-functional effects induced by 6-month treatment with OS-Citicoline would persist after a 3-month period of treatment suspension.

## Materials and methods

### Subjects

Forty volunteer patients (25 females and 15 males) affected exclusively in one eye by NAION (mean age, 59.61±7.21 years) and 20 age-matched (12 females and 8 males) normal control subjects (mean age, 60.20±7.32 years) participated in the study.

All subjects underwent extensive ophthalmologic evaluation, including: best-corrected visual acuity (VA), slit-lamp biomicroscopy, intraocular pressure (IOP) measurement, indirect ophthalmoscopy, optic nerve head 30° colour standard photography, Humphrey 24–2 automated visual field test (HFA 24–2), OCT, PERG, and VEP simultaneous recordings.

Inclusion criteria for NAION patients were: age > 45 years; disease persisting between 6 and 12 months from an episode of sudden, painless, unilateral visual loss accompanied by an afferent pupil defect and/or acquired dischromatopsia [54,55]; visual acuity > 0.8 LogMAR and visual field defects (diffuse/altitudinal scotoma) by HFA 24–2; IOP<18 mmHg; no abnormalities of the anterior segment in both eyes; exclusion of clinical and laboratory data leading to Horton disease; history or presence of any other type of optic neuropathy (demyelinating, inflammatory, toxic, or hereditary); absence of optic disc oedema or RNFL swelling (evaluated by OCT, see below); presence of ophthalmoscopic sign of pale optic disc head; absence of fluorangiographic sign of any type of retinal vasculopathy (i.e., central vein/artery occlusion); absence of intake of drugs with potential neuroprotective effects (i.e., topical brimonidine tartrate and coenzyme-Q10 drops [56,57]) for at least 12 months prior to enrolment.

For both NAION patients and control subjects, exclusion criteria were: presence of moderate to dense lens opacities or maculopathy which are known to affect PERG and VEP responses [58,59], presence of corneal opacities, previous history of refractive surgery, glaucoma, or ocular hypertension, intraocular inflammation such as anterior or posterior uveitis, retinal detachment, or laser treatment for peripheral retinal diseases, ocular trauma, diabetes, and other systemic or neurological diseases.

Excluded from the present study were all NAION patients with visual field centrocecal scotoma that did not allow perception of the target of PERG and VEP stimulation (see below).

### Study design

The present study was initially not registered at ClinicalTrials.gov before the enrolment of participants as we erroneously believed that Citicoline is a “dietary supplement”, and studies regarding effects of dietary supplements did not require registration. Subsequently, we rectified this error and initiated an actual registration at ClinicalTrials.gov (NCT03758118). The authors confirm that all ongoing and related trials for this drug/intervention are registered. This study was designed as a randomized, monocentric, prospective, and operator-masked study. The research followed the tenets of the Declaration of Helsinki, and the study was approved by the local ethics committee on February 14^th^, 2017 (Comitato Etico Centrale IRCCS Lazio, Sezione IFO/Fondazione Bietti, Rome, Italy). Upon recruitment, executed from February to July 2017, at the IRCCS-Fondazione Bietti, each patient signed informed consent. 1) Baseline. All enrolled NAION patients were randomly divided into two age-similar Groups comprising 20 patients each: the NN Group (20 enrolled eyes; mean age 60.57±7.29 years) and NC Group (20 enrolled eyes; mean age 58.29±5.87 years). The separation of NAION untreated and NAION-treated with OS-Citicoline patients (screened by VP and LB) was performed by an electronically generated randomization system that divided all NAION patients into two above- mentioned groups on the basis of similar age, equal distribution of males and females, and similar HFA visual defects. The key was opened to all investigators at the end of the follow-up period.

**Fig 1:**
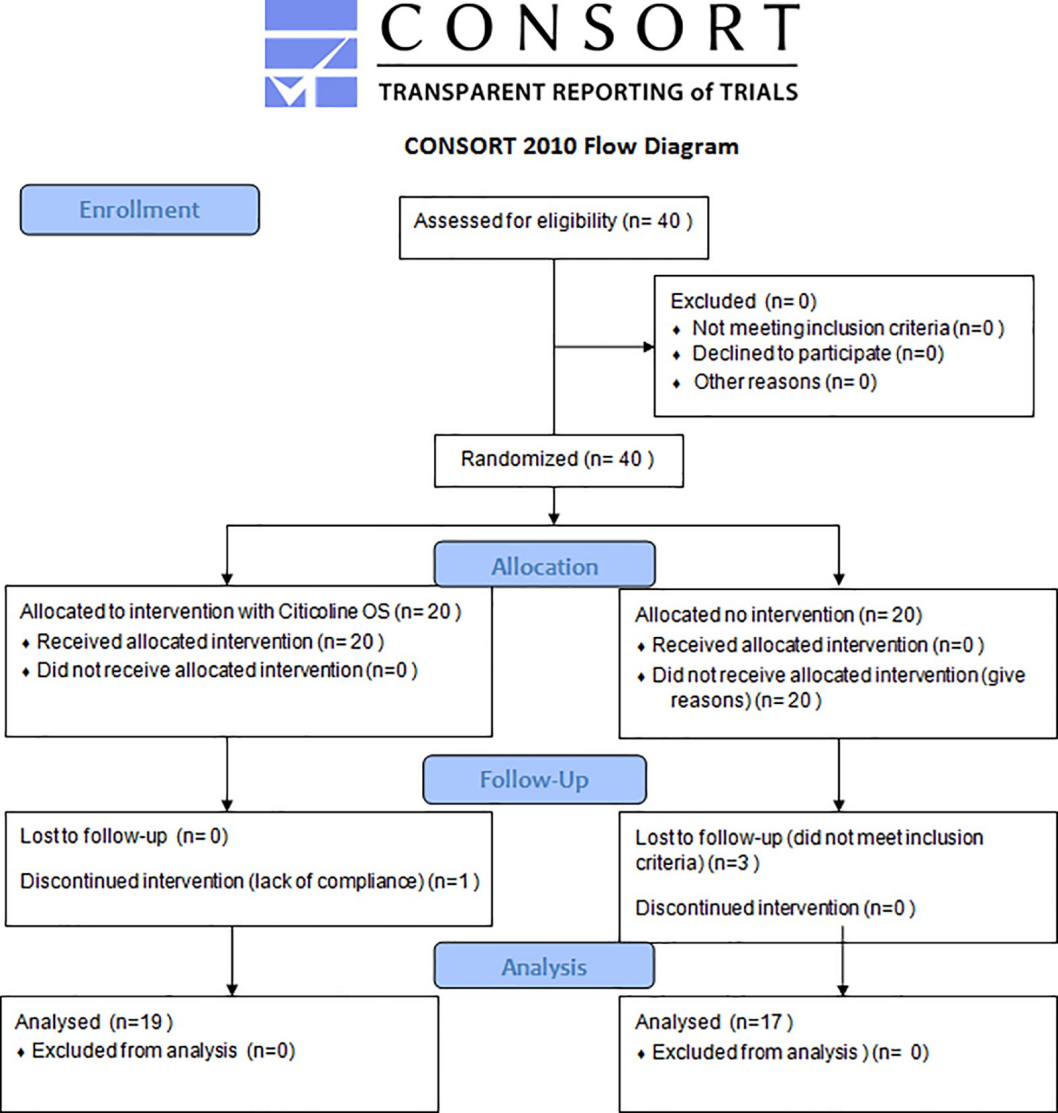
CONSORT participant flow diagram.

2) Months 0–6. Throughout a 6-month period, no treatment was performed in the NN Group, while the NC Group received one vial of OS-Citicoline containing 500 mg of Citicoline (Neukron Ofta, Omikron Italia, Italy) per day. During this period, one eye belonging to the NC Group was excluded for lack of compliance. Therefore, 19 NC eyes out of 20 eyes (from 12 female and 7 male patients, mean age 59.96±8.38 years) were assessed in the study. Three eyes belonging to the NN Group were excluded on the basis of IOP increase (>21 mmHg and <24 mmHg). Therefore, 17 NN eyes out of 20 eyes (from 11 female and 6 male patients, mean age 58.63±6.02 years) were assessed in the study.

3) Months 6–9. After a 6-month period of treatment with OS-Citicoline, a 3-month period of wash-out was performed in the NC Group. No other treatment was applied. This protocol was similar for the NN Group. The CONSORT Participant Flow Diagram is reported in Fig 1.

Following previously published criteria [39,41,60], in order to evaluate PERG and VEP responses, OCT, HFA, and VA data independently from clinical conditions and patient groups, all examinations (electrophysiological, RNFL-T, HFA, and VA measurements) were performed at baseline and after 6 and 9 months of follow-up in the presence of four operators (GC, LZ, FP, and VVF, see Acknowledgments), who were blinded to patient Groups.

At month 6, compliance to Citicoline administration was assessed by evaluating the ratio between the number of OS-Citicoline vials delivered at baseline and that returned from each NC patient at the 6-month evaluation. A ratio > 90% was considered “good compliance”. This value was attained by all but one of the NC enrolled patients.

### Visual Acuity assessment

Best-corrected VA was evaluated by the modified Early Treatment Diabetic Retinopathy Study (ETDRS) Tables (Lighthouse, Low Vision Products, Long Island City, NY, USA) at the distance of 4 meters. VA was measured as a logarithm of minimum angle of resolution (LogMAR) values.

### Electrophysiological examinations

In Controls, NN, and NC eyes, electrophysiological examinations were performed at baseline, and after 6 and 9 months of follow-up in NN and NC eyes.

In accordance with our previously published studies [11,38,39,41,60,61], simultaneous PERG and VEP recordings were performed using the methods briefly described below.

Subjects were seated and adapted to room light in a semi-dark, acoustically isolated room for 10 minutes in front of the display and surrounded by a uniform field of luminance of five candelas per m^2^. Pupil diameter was approximately 5 mm. No mydriatic or miotic drugs were used. Stimulation was monocular after occlusion of the other eye. Visual stimuli were checkerboard patterns (contrast, 80%; mean luminance, 110 cd/m^2^) generated on a TV monitor and reversed in contrast at the rate of two reversals per second. At the viewing distance of 114 cm, the check edges subtended 60 minutes (60’) and 15 minutes (15’) of the visual angle. We used two different checkerboard patterns as suggested by the ISCEV standards [62] to obtain a prevalent activation of larger (60’ checks) or smaller (15’ checks) axons. [61,63–66] The monitor screen subtended 23°. A small fixation target, subtending a visual angle of approximately 0.5° (estimated after taking into account spectacle-corrected individual refractive errors), was placed at the center of the pattern stimulus. For every PERG and VEP acquisition, each patient positively reported that he/she could clearly perceive the fixation target.

The characteristics of PERG and VEP recordings have been reported extensively in our previously published studies [11,38,39,41,60,61].

In the analysis of PERG recordings, we considered the P50-N95 peak-to peak amplitude (PERG-A). In the analysis of VEP recordings, we considered the P100 implicit time (VEP-IT) and the N75-P100 peak-to peak amplitude (VEP-A).

### Visual field analysis

Visual fields (HFA, protocol Sita Standard 24–2; Zeiss, San Leandro, CA, USA) were performed in Controls, NC, and NN patients at baseline and twice at each evaluation (baseline, 6 and 9 months). The second examination was considered for analysis. The mean deviation (MD) and corrected pattern standard deviation (CPSD) indexes of the HFA were considered.

### Optical coherence tomography analysis

In Controls, NC, and NN Groups, RNFL-T was assessed using spectral domain OCT (RTVue Model-RT100 version 3.5; Optovue Inc, Fremont, CA, USA). Peripapillary RNFL 3.45 protocol was used. The characteristics of OCT evaluation have been reported extensively in our previous work [67].

In the OCT results, we considered the average value of RNFL-T of four measurements per quadrant: superior (RNFL-TS), inferior (RNFL-TI), nasal (RNFL-TN), and temporal (RNFL-TT); the overall data obtained in all quadrants (average of 16 values) was identified as RNFL Overall (RNFL-TO).

### Statistics

Sample size estimates were obtained from pilot evaluations performed in 12 eyes from 12 NAION eyes, and 12 eyes from 12 control subjects, other than those included in the current study (unpublished data). Inter-individual variability, expressed as standard deviation (SD) data, was estimated for 15’ PERG-A. For PERG-A, SD values were significantly higher for patients (mean: 1.45 microvolt; SD: 0.43 microvolt, about 30% of the mean) than for controls (mean: 2.23 microvolt; SD: 0.34 microvolt, about 15% of the mean).

It was also established that, assuming the above between-subjects SD, sample sizes of control subjects and patients belonging to the NAION Group provided a power of 95% (β = 5%) at α = 1% for detecting a between-group difference of 34% in PERG-A measurement. Assuming a drop out value of 30%, a sample size of 15 patients and 15 control subjects was obtained.

These changes were expected to be clinically meaningful when comparing results of treated NAION eyes observed at baseline conditions versus those observed at 6 and 9 months of follow-up.

Test-retest data (obtained in the Group of NAION eyes evaluated in this study) of PERG, VEP and RNFL-T results were expressed as the mean difference between two recordings obtained in separate sessions ± SD of this difference. A 95% confidence limit (CL, mean ± 2 SD) of test-retest variability in NAION eyes was established assuming a normal distribution.

At baseline, absolute values of VA, PERG and VEP responses, RNFL-T, and HFA MD values observed in NC and NN eyes were compared between Groups and Controls by repeated measures one-way analysis of variance (ANOVA).

During the follow-up, the differences in VA, PERG, VEP, RNFL-T, and HFA MD values observed in individual NAION eyes with respect to baseline values (values detected at 6 and 9 months minus those detected at baseline) were calculated by performing a logarithmic transformation to better approximate a normal distribution. The statistical significance of the mean of individual differences of these parameters detected in NN and NC Groups were evaluated by ANOVA. The changes in absolute values of PERG, VEP, and RNFL-T with respect to baseline were evaluated by ANOVA separately for each Group.

Pearson’s correlation was used to assess the relationship between the differences (logarithmic values at 6 and 9 months minus logarithmic values at baseline) in electrophysiological (PERG and VEP), morphological (RNFL-T), and HFA data.

All statistical analyses were performed using MedCalc V.13.0.4.0 (MedCalc, Mariakerke, Belgium). A p-value less than 0.01 was considered as statistically significant for all analyses.

## Results

**Fig 2:**
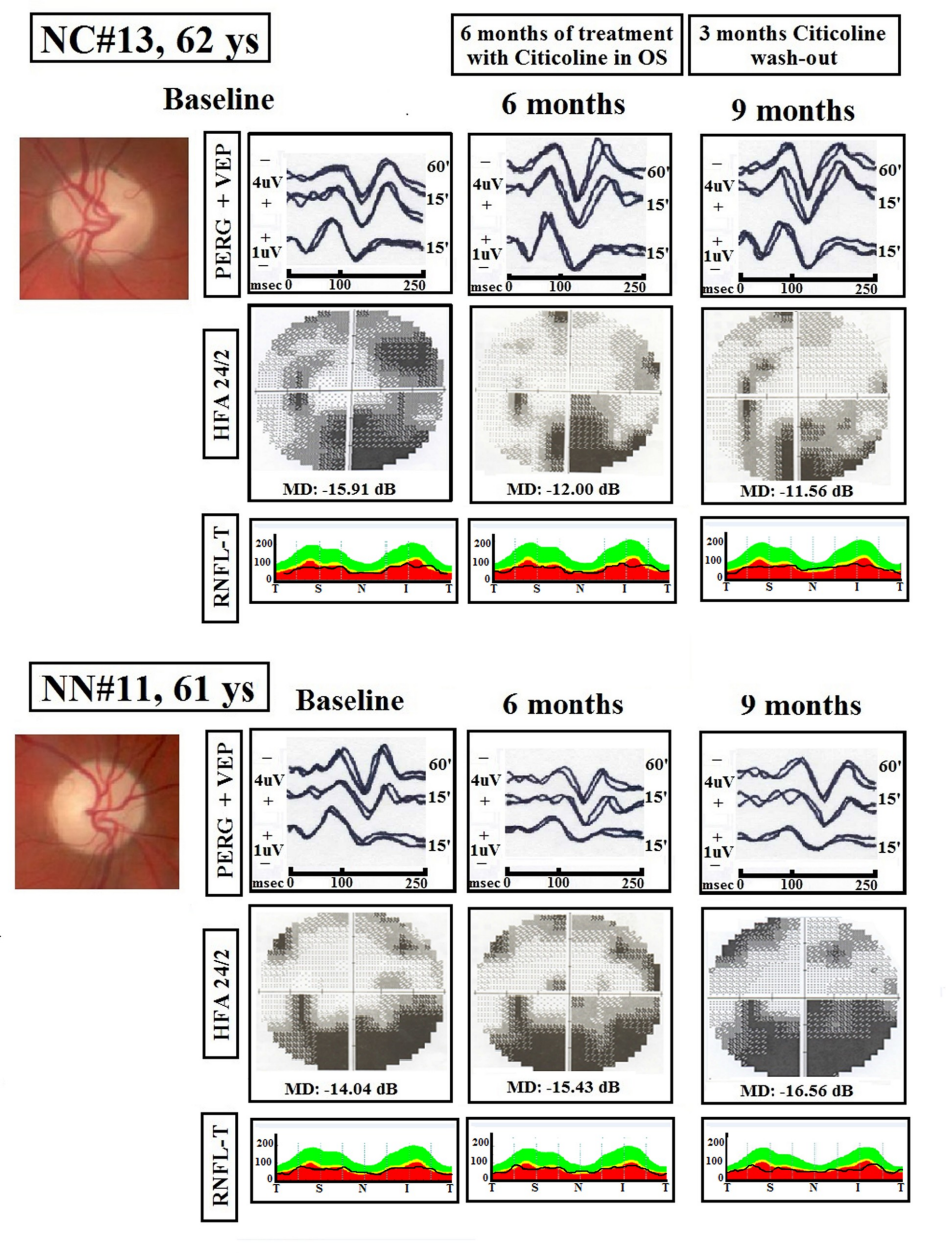
Examples of simultaneous Visual Evoked Potentials (VEP) and Pattern Electroretinogram (PERG) recordings, Humphrey Field Analyzer (HFA 24–2) and Retinal Nerve Fiber Layer Thickness (RNFL-T) by optical coherence tomography (OCT) from two patients with non-arteritic ischemic optic neuropathy (NAION).

Fig 2 presents examples of simultaneous PERG and VEP recordings, and relative HFA and RNFL-T from one representative NAION patient treated with OS-Citicoline for 6 months followed by a 3–month period of Citicoline wash-out (NC#13), and from one NAION patient in which no treatment was performed during a 9-month period of follow-up (NN#11).

One NAION patient was treated with Citicoline in oral solution (OS-Citicoline) during a 6-month period followed by a 3-month period of wash-out (NC#13 eye); one NAION patient was not treated for a total period of 9 months (NN#11 eye).

60’ and 15’ refer to visual stimuli in which each check subtended 60 and 15 minutes of the visual arc, respectively. MD refers to Mean Deviation of HFA 24–2.

At baseline for both NC and NN eyes, the optic nerve head with a similar diffuse pallor was displayed. Compared to baseline condition, after treatment with OS-Citicoline (6 months), an increase in PERG amplitudes, shortening of 60’ and 15’ VEP implicit times, increase of 60’ and 15’ VEP amplitudes, reduction of MD, and unmodified RNFL-T was observed in NC eyes. At the end of wash-out, electrophysiological, HFA, and RNFL-T findings were similar to those observed at the end of treatment. By contrast, at both 6 and 9 months of follow-up, a reduction in PERG amplitudes, increase of 60’ and 15’ VEP implicit times, and reduction of 60’ and 15’ VEP amplitudes were associated with a worsening of MD and RNFL-T in NN eyes.

### Baseline

Considering the mean values observed in the NN and NC Groups, for each study parameter, a significant difference (p<0.01) was detected between Groups compared to Controls; no significant (p>0.01) differences were observed when comparing NN and NC Groups.

Mean data of PERG, VEP, RNFL-T, HFA, and VA parameters observed in Controls, NN, and NC Groups at baseline, relative statistical analysis of Groups, and the number of normal or abnormal NN and NC eyes are reported in Table 1.

**Table 1:** Mean values of age, Pattern Electroretinogram (PERG) P50-N95 amplitudes, Visual Evoked Potentials (VEP) P100 implicit times, N75-P100 amplitudes, Retinal Nerve Fiber Layer (RNFL) thickness, Humphrey 24–2 perimetry (HFA) Mean Deviation (MD), and LogMAR Visual Acuity (VA) detected at baseline in Controls (C, N = 20 eyes), in patients with Non-Arteritic Ischemic Optic Neuropathy (NAION) treated with Citicoline in oral solution (NC Group, N = 19 eyes) and in untreated NAION patients (NN Group, N = 17 eyes).

	Group	Mean	SD	ANOVA: NC vs C: f(1,38); NN vs C: f(1,36);		ANOVA: NN vs NC: f(1,35);		Nr	Ab
				f =	P =	f =	P =		
**Age (years)**	**C**	60.20	7.32						
	**NN**	58.13	6.02	0.50	0.486				
	**NC**	59.96	8.38	0.04	0.837	0.29	0.592		
**60’ PERG P50-N95 A (μV)**	**C**	2.41	0.14						
	**NN**	1.52	0.23	209.02	<0.001			0	17
	**NC**	1.34	0.36	152.58	<0.001	3.11	0.087	0	19
**60’VEP P100 IT (msec)**	**C**	100.51	3.37						
	**NN**	126.71	5.87	287.81	<0.001			0	27
	**NC**	127.52	7.86	198.08	<0.001	0.12	0.731	0	19
**60’ VEP N75-P100 A (μV)**	**C**	12.43	1.88						
	**NN**	5.36	2.84	80.44	<0.001			1	16
	**NC**	4.02	2.66	131.09	<0.001	2.14	0.153	2	17
**15’ PERG P50-N95 A (μV)**	**C**	2.52	0.19						
	**NN**	1.38	0.28	215.42	<0.001			0	17
	**NC**	1.32	0.39	151.63	<0.001	0.28	0.603	0	19
**15’VEP P100 IT (msec)**	**C**	103.60	3.86						
	**NN**	126.88	6.50	181.74	<0.001			0	17
	**NC**	127.05	8.35	128.09	<0.001	0.00	0.947	0	19
**15’ VEP N75-P100 A (μV)**	**C**	11.34	1.72						
	**NN**	5.87	2.53	60.67	<0.001			3	14
	**NC**	4.64	2.64	89.09	<0.001	2.03	0.164	2	17
**RNFL-TO (μ)**	**C**	113.92	4.61						
	**NN**	63.99	8.44	519.46	<0.001			0	17
	**NC**	57.71	9.43	568.27	<0.001	4.39	0.044	0	19
**RNFL-TS (μ)**	**C**	132.30	11.24						
	**NN**	69.91	9.25	332.12	<0.001			0	17
	**NC**	61.89	14.02	300.96	<0.001	4.00	0.054	0	19
**RNFL-TN (μ)**	**C**	96.42	9.15						
	**NN**	54.59	13.91	120.08	<0.001			0	17
	**NC**	46.26	9.29	288.49	<0.001	4.55	0.040	0	19
**RNFL-TI (μ)**	**C**	140.51	10.86						
	**NN**	76.95	16.72	193.53	<0.001			1	16
	**NC**	75.84	21.34	144.45	<0.001	0.03	0.864	2	17
**RNFL-TT (μ)**	**C**	86.42	8.62						
	**NN**	54.53	16.17	58.46	<0.001			2	15
	**NC**	46.84	10.32	169.66	<0.001	2.96	0.095	0	19
**HFA MD (dB)**	**C**	0.23	0.62						
	**NN**	-13.02	7.15	68.42	<0.001			0	17
	**NC**	-15.61	6.43	120.36	<0.001	1.31	0.260	0	19
**VA (LogMAR)**	**C**	0.00	0.00						
	**NN**	0.18	0.24	11.31	0.002			7	10
	**NC**	0.17	0.25	9.26	0.004	0.01	0.904	7	12

ANOVA: One-way Analysis of Variance. SD: 1 standard deviation. 60’ and 15’: visual stimuli in which each check subtended 60 and 15 minutes of the visual arc, respectively. A, Amplitude; μV, microvolt; IT, Implicit Time; msec, milliseconds; TO, Overall Thickness; TS, Superior Thickness; TN, Nasal Thickness; TI, Inferior Thickness; TT, Temporal Thickness; μ, microns; LogMAR, logarithm of the minimum angle of resolution. Nr, number of eyes inside the normal limits. Ab, number of eyes outside the normal limits. Normal limits were obtained from control subjects by calculating mean values +2 standard deviations for VEP P100 implicit time and mean values –2 standard deviations for PERG P50-N95, VEP N75-P100 amplitudes, and RNFL thickness. MD was considered as Ab for values less than -2 dB. VA was considered as Ab for values greater than 0.0.

### Months 0–6: 6-month treatment with OS-Citicoline in NC eyes and 6-month follow-up in NN eyes

#### Functional assessment: PERG and VEP data

The individual changes in 95% CL of 60’ and 15’ PERG-A, VEP-IT, and VEP-A after 6 months of follow-up are reported in “S1 Table”. In the NC Group, the mean of individual changes of electrophysiological parameters was significantly different (p<0.01) compared to that in the NN Group. Mean individual differences (6 months minus baseline) and relative statistical analyses between Groups are reported in Table 2.

**Table 2:** Mean values of the individual differences (6 months minus baseline and 9 months minus baseline) in Pattern Electroretinogram (PERG) P50-N95 Amplitudes, Visual Evoked Potentials (VEP) P100 Implicit times and N75-P100 Amplitudes, Retinal Nerve Fiber Layer (RNFL) thickness, in Humphrey 24–2 perimetry (HFA) mean deviation (MD), and logMAR visual acuity (VA) observed in patients with non-arteritic ischemic optic neuropathy (NAION) treated with Citicoline in oral solution (NC Group, N = 19 eyes) and in untreated NAION patients (NN Group, N = 17 eyes).

	Group NN (N = 17)		Group NC (N = 19)		ANOVA vs NN	
	Mean	SD	Mean	SD	F (1,35)	P =
**Difference in 60’ PERG P50-N95 A (log μV)**						
**6 months minus baseline**	-0.0822	0.0651	0.1135	0.0602	87.82	<0.0001
**9 months minus baseline**	-0.1208	0.0614	0.1251	0.0678	128.71	<0.0001
**Difference in 60’VEP P100 IT (log msec)**						
**6 months minus baseline**	0.0185	0.0132	- 0.0249	0.0145	87.09	<0.0001
**9 months minus baseline**	0.0246	0.0106	- 0.0315	0.0208	100.02	<0.0001
**Difference in 60’ VEP N75-P100 A (log μV)**						
**6 months minus baseline**	-0.1051	0.0595	0.2600	0.2727	29.12	<0.0001
**9 months minus baseline**	-0.1762	0.1090	0.2141	0.1971	52.08	<0.0001
**Difference in 15’ PERG P50-N95 A (log μV)**						
**6 months minus baseline**	-0.0911	0.0914	0.1252	0.0988	46.07	<0.0001
**9 months minus baseline**	-0.1232	0.0919	0.1123	0.1183	43.47	<0.0001
**Difference in 15’VEP P100 IT (log msec)**						
**6 months minus baseline**	0.0156	0.00889	-0.0283	0.01425	119.58	<0.0001
**9 months minus baseline**	0.0219	0.01232	-0.0300	0.0173	105.18	<0.0001
**Difference in 15’ VEP N75-P100 A (log μV)**						
**6 months minus baseline**	-0.0891	0.0651	0.1025	0.0872	54.68	<0.0001
**9 months minus baseline**	-0.2358	0.1474	0.135	0.189	42.18	<0.0001
**Difference in RNFL-TO (log μ)**						
**6 months minus baseline**	-0.0510	0.0421	0.0552	0.0529	43.68	<0.0001
**9 months minus baseline**	- 0.0898	0.0728	0.0599	0.0514	51.72	<0.0001
**Difference in RNFL-TS (log μ)**						
**6 months minus baseline**	-0.0506	0.0656	0.0858	0.0852	28.42	<0.0001
**9 months minus baseline**	-0.0902	0.1126	0.0913	0.0769	32.49	<0.0001
**Difference in RNFL-TN (log μ)**						
**6 months minus baseline**	-0.0451	0.0490	0.0405	0.0669	18.79	0.0001
**9 months minus baseline**	-0.0739	0.0784	0.0594	0.0607	32.91	<0.0001
**Difference in RNFL-TI (log μ)**						
**6 months minus baseline**	-0.0457	0.0507	0.0552	0.0529	30.41	<0.0001
**9 months minus baseline**	- 0.0729	0.0854	0.0702	0.0495	38.82	<0.0001
**Difference in RNFL-TT (log μ)**						
**6 months minus baseline**	-0.1112	0.1035	0.0160	0.0989	14.19	0.0006
**9 months minus baseline**	-0.1153	0.1590	0.0077	0.1100	7.32	0.0100
**Difference in HFA 24–2 MD (dB)**						
**6 months minus baseline**	-2.62	2.41	2.07	1.87	43.05	<0.0001
**9 months minus baseline**	-2.93	2.11	1.93	1.93	52.11	<0.0001
**Difference in VA (logMAR)**						
**6 months minus baseline**	0.0412	0.0819	-0.0468	0.0852	9.93	0.0034
**9 months minus baseline**	0.0535	0.0914	-0.0574	0.1147	10.11	0.0031

ANOVA: One-way analysis of variance between NC and NN eyes. SD: 1 standard deviation. 60’ and 15’: visual stimuli in which each check subtended 60 and 15 minutes of the visual arc, respectively; A, Amplitude; μV, microvolt; IT, implicit time; msec, milliseconds; TO, Overall Thickness; TS, Superior Thickness; TN, Nasal Thickness; TI, Inferior Thickness; TT, Temporal Thickness; μ, micron; N, number of eyes.

**Fig 3:**
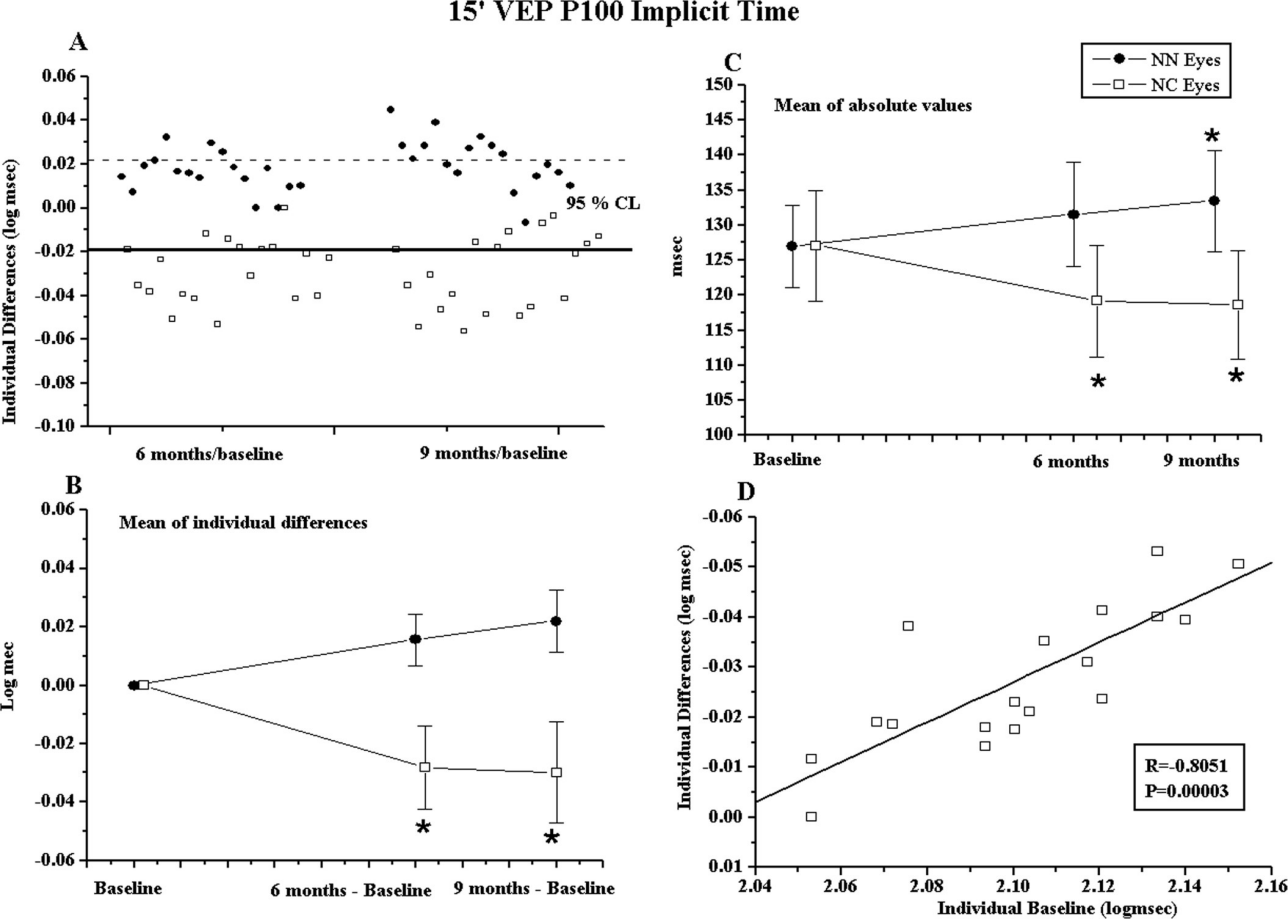
Pattern and visual evoked potentials (VEP) P100 implicit time recorded in response to 15’ checks (15’) results. **(A)** Mean of absolute PERG P50-N95 Amplitude values observed in NC and NN Groups. * = ANOVA, p<0.01 in NN and NC Groups with respect to baseline. Vertical lines: one mean standard deviation. The statistical evaluation is reported in S2 Table. **(B)** Individual PERG P50-N95 Amplitude values observed in NC eyes at baseline plotted as a function of the values of the corresponding differences at the end of treatment (6 months minus baseline). Pearson’s test was used for regression analysis and linear correlation. **(C)** Mean of absolute VEP P100 implicit time values observed in NC and NN Groups. * = ANOVA, p<0.01 in NN and NC Groups with respect to baseline. Vertical lines: one mean standard deviation. Statistical evaluation is reported in “S2 Table”. **(D)** Individual VEP P100 implicit time values observed in NC eyes at baseline plotted as a function of the values of the corresponding differences at the end of treatment (6 months minus baseline). Pearson’s test was used for regression analysis and linear correlation.

In NC eyes, positive changes in individual 60’ and 15’ PERG-A, VEP-IT, and VEP-A at 6 months follow-up was significantly linearly correlated (p<0.01) with greater impairment at baseline (Fig 3B for 15’ PERG-A and Fig 3D for 15’ VEP-IT). The 60’ and 15’ VEP-IT shortening was not significantly correlated (p>0.01) with relative changes in PERG-A (“S1A and S1B Fig”).

In the NC Group, the means of absolute values of 60’ and 15’ PERG-A and VEP-IT were significantly increased and reduced (p<0.01), respectively, when compared to that at baseline. No significant differences (p>0.01) in 60’ and 15’ VEP-A were observed. In the NN Group, no significant changes (p>0.01) in mean values of 60’ and 15’ VEP parameters were observed, while a significant reduction (p<0.01) in 60’ and 15’ PERG-A was observed. Mean data of absolute values of PERG and VEP parameters observed in NN and NC Groups at baseline, after 6 and 9 months, and relative statistical analyses with respect to baseline are shown in “S2 Table” and in Fig 3A and 3C for 15’ PERG-A and 15’ VEP-IT, respectively.

#### Morphological assessment: OCT data

**Fig 4:**
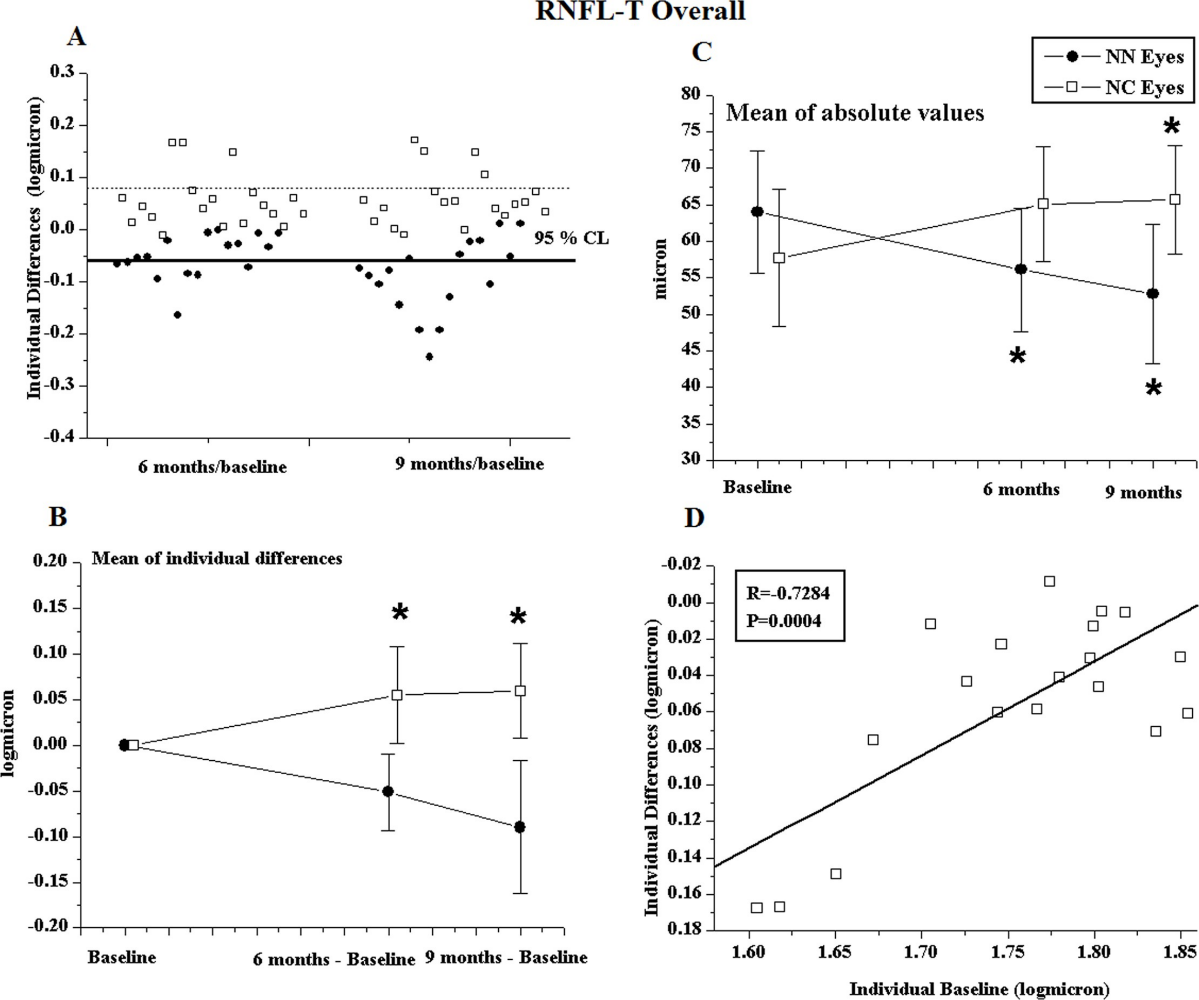
Retinal Nerve Fiber Layer Thickness average from all quadrants (RNFL-T Overall) results. **(A)** Individual changes detected in patients with Non-Arteritic Ischemic Optic Neuropathy (NAION) treated with Citicoline in oral solution (NC Group, N = 19 eyes) and in untreated NAION patients (NN Group, N = 17 eyes). The percentage of unmodified eyes (within the 95% confidence test-retest limit), eyes with improvement (values over the 95% confidence test-retest limit, dashed line), and eyes with worsening (values under the 95% confidence test-retest limit, solid line) are reported in “S1 Table”. **(B)** Mean of individual differences observed in NC and NN Groups. * = ANOVA, p<0.01 between NN and NC Groups. Vertical lines: one mean standard deviation. Statistical evaluation is reported in Table 2. **(C)** Mean of absolute values observed in NC and NN Groups. * = ANOVA, p<0.01 in NN and NC Groups with respect to baseline. Vertical lines: one mean standard deviation. The statistical evaluation is reported in “S3 Table”. **(D)** Individual values observed in NC eyes at baseline condition plotted as a function of the values of the corresponding differences at the end of treatment (6 months minus baseline). Pearson’s test was used for regression analysis and linear correlation.

In the NC Group, a large percentage of eyes showed unmodified RNFL-T (ranging from 42.11% of RNFL-TT to 78.95% of RNFL-TN), a small percentage of eyes presented an increase of RNFL-T (ranging from 21.05% of RNFL-TN to 47.37% of RNFL-TS), and a smaller percentage of eyes showed a reduction in RNFL-T (ranging from 0% of RNFL-TN to 31.6% of RNFL-TT). The number of individual changes expressed in absolute values and percentages with respect to the total number of eyes belonging to NN and NC Groups at months 6 and 9 of follow-up are presented in “S1 Table”. Individual changes in RNFL-TO observed in NN and NC eyes at 6 and 9 months compared to baseline are shown in Fig 4A.

In individual NC eyes, positive changes in RNFL-TO were significantly linearly correlated (p<0.01) with greater impairment at baseline (see Fig 4D) but were not significantly linearly correlated (p>0.01) with relative changes in 60’ and 15’ PERG and VEP parameters. The correlations between RNFL-TO and 60’ VEP-IT, and RNFL-TT and 15’VEP-IT are reported in “S1C and S1D Fig”, respectively.

In the NN Group, a high percentage of eyes showed a reduction in RNFL-T (ranging from 29.41% of RNFL-TI to 52.94% of RNFL-TT) (see “S1 Table” “6 months minus baseline difference” and Fig 3A). The mean of individual changes of RNFL-T was significantly different (p<0.01) from the NC Group (see Table 2 “6 months minus baseline difference” and Fig 4B for RNFL-TO).

On average, when compared to baseline, in the NC Group, the mean of absolute values of RNFL-TN, TI, and TT were not significantly altered (p>0.01), while the absolute values of RNFL-TO and TS were significantly increased (p<0.01). In the NN Group, mean absolute values of RNFL-TO and TT were significantly reduced (p<0.01), while mean absolute values of RNFL-TS, TN, and TI were not significantly changed (p>0.01). Mean data of absolute values of RNFL-T parameters observed in NN and NC Groups at baseline and after 6 months, and relative statistical analyses with respect to baseline are shown in “S3 Table”, and those for RNFL-TO are shown in Fig 4C.

#### Psychophysical assessment: VA and HFA data and their correlation with functional and morphological data

VA was reduced in a small percentage of NC eyes (5.27%), while five NN eyes (29.41%) showed a decrease in visual acuity. The number of individual changes is presented in “S1 Table”. On average, individual VA changes in the NC Group were significantly (p<0.01) different with respect to those in the NN Group (see Table 2, “6 months minus baseline”).

Considering individual HFA MD values with an increase/decrease greater than 1.0 dB with respect to baseline, in several NC eyes (14 out of 19, 73.68%) an improvement of visual field defects was observed. As a consequence, the individual increment in MD induced a positive mean progressive rate (2.07 dB). Individual MD changes in the NC Group were not significantly correlated (p>0.01) with the changes in 60’ and 15’ PERG-A or RNFL-T. The increase in VA and MD was significantly correlated (p<0.01) with the shortening of both 60’ and 15’ VEP-IT values. The linear correlations between VEP and MD differences are presented in “S1E and S1F Fig”.

In the NN Group, a worsening of MD values was observed in 12 (70.59%) eyes with consequent mean progressive rate of -2.62 dB. On average, in the NC Group, individual changes in HFA MD were significantly different (p<0.01) with respect to those in the NN Group (see Table 2, “6 months minus baseline”).

### Month 9: 3-month period of OS-Citicoline wash-out in NC eyes and 9- month follow-up in NN eyes

#### Functional assessment: PERG and VEP data

The individual changes observed after 9 months of follow-up are reported in S1 Table. In the NC Group, the mean of individual changes was significantly different (p<0.01) compared to that observed in the NN Group. Mean individual differences (9 months minus baseline) and relative statistical analyses between Groups are reported in Table 2.

Considering the mean changes of absolute values of PERG and VEP parameters with respect to baseline, in the NC Group, we observed that 60’ PERG-A, and 60’ and 15’ VEP-IT were still significantly increased and reduced (p<0.01), respectively. In the NN Group, we detected a significant reduction (p<0.01) in mean values of 60’ and 15’ PERG-A and a significant increase (p<0.01) of 60’ and 15’ VEP-IT (see “S2 Table” “9 months” and Fig 3A for 15’ PERG-A, and Fig 3C for 15’ VEP-IT).

#### Morphological assessment: OCT data

In the NC Group, the majority of eyes showed unmodified RNFL-T (ranging from 41.18% of RNFL-TT to 82.35% of RNFL-TI). Only a few eyes showed increased RNFL values (ranging from 29.41% of RNFL-TI to 47.06% of RNFL-TS) and no eyes (0%) showed a reduction in RNFL-TO, TS, TN, and TI, while only six eyes (32.29%) presented a reduction in RNFL-TT. In the NN Group, a high percentage of eyes showed a reduction in RNFL-T (ranging from 29.41% of RNFL-TI to 64.71% of RNFL-TT) (see “S1 Table” “9 months minus baseline difference” and Fig 4A).

On average, in the NC Group, the mean individual changes in RNFL-T were significantly (p<0.01) different from NN Group ones (see Table 2 “9 months minus baseline” and Fig 4B).

When compared to baseline, in the NC Group, the mean of absolute values of RNFL-TN, TI, and TT was not significantly altered (p>0.01), while the mean of absolute values of RNFL-TO and TS was significantly increased (p<0.01). In the NN Group, the mean of absolute values of RNFL-TO, TS, and TT was significantly reduced (p<0.01), while the mean of absolute values of RNFL-TN and TI was not significantly modified (p>0.01) (see “S3 Table” “9 months” and Fig 4C for RNFL-TO).

#### Psychophysical assessment: VA and HFA data and their correlation with functional and morphological data

In the NC Group, VA was still reduced in one eye (5.26%), while seven NN eyes (41.18%) showed a decrease in VA. Therefore, the differences in VA detected in the NC Group were significantly (p<0.01) higher than those in the NN Group ones (see Table 2, “9 months minus baseline”).

In 12 (70.59%) NC eyes, an improvement of visual field defects was observed, while in 13 (76.47%) NN eyes, a further worsening of visual field defects was observed. Therefore, in NC eyes, there was a positive mean progressive rate of 1.93 dB that was significantly different (p<0.01) from the negative mean progressive rate of -2.93 observed in NN eyes (see Table 2, “9 months minus baseline”).

Throughout the entire period of treatment with OS-Citicoline and after the 3-month period of Citicoline wash-out, no adverse side effects were reported from all patients enrolled in the study.

## Discussion

The aim of our study was to evaluate whether treatment with Citicoline in oral solution would induce an increase in visual function, RGCs function, neural conduction along visual pathways (*neuroenhancement)*, preservation of RGCs fibers (*neuroprotection*), or both, using a human model of neurodegeneration such as NAION.

Our results demonstrated that along a 9-month period of follow-up, significant differences between NAION untreated (NN eyes) and NAION OS-Citicoline-treated (NC eyes) patients were observed. RGCs function (evaluated by PERG recording) increased in NC and worsened in NN. Neural conduction along the large and small axons forming the visual pathways (evaluated by VEP recordings) improved in NC with consequent increase in visual acuity and perimetric index (HFA-MD) but were further delayed in NN. RGCs fibers’ morphology (evaluated by RNFL thickness) stabilized/improved in NC and further worsened in NN.

Collectively, these results suggested that in this model of optic nerve neurodegeneration (NAION), both neuroenhancer and neuroprotective effects were present following treatment with OS-Citicoline. Neuroenhancer and neuroprotective effects are separately discussed as follows.

### Neuroenhancement: PERG data, VEP data, and their correlation with psychophysical (VA and HFA) data

#### Retinal ganglion cells function: PERG data

PERG-A is an electrophysiological parameter that reflects the bioelectrical activity of the innermost retinal layers (ganglion cells and their fibers). [68] At baseline (Table 1), we found significant RGCs dysfunction (reduced PERG-A) in both treated and untreated NAION eyes when compared to that of Controls; however, they were similar in both Groups. After 6 months (Table 2 and “S2 Table”), while PERG-A was significantly reduced in NN eyes, it was significantly increased in the NC Group compared to baseline. By considering the mean of individual differences compared to baseline, this functional recovery remained significant after 3 months of OS-Citicoline wash-out in NC eyes, whereas NN eyes continued to show a significant reduction in PERG-A, possibly due to impaired blood supply or degenerative processes. Our results in NC eyes are in agreement with published evidence describing short-term favourable effects of Citicoline in oral suspension [41] in reducing retinal dysfunction, possibly due to its ability to limit structural phospholipid degradation of cellular membranes [29] and to reinforce dopaminergic transmission to the retina [48]. Similar effects of Citicoline administered in various modalities (intramuscular, oral suspension, and eye drops) and a relative increase in RGCsfunction in another model of optic nerve degeneration (open angle glaucoma) have been extensively reported in our recent review [45].

#### Neural conduction along visual pathways: VEP data and their correlation with psychophysical (VA and HFA) data

VEP in response to 60’ and 15’ checks are bioelectrical responses of the visual cortex that provide information about the function of the large and small axons forming the visual pathways [61,67].

In NN eyes, we observed a significant increase of VEP-IT and reduction of VEP-A during 9 months of follow-up (“S2 Table”). This suggests a progressive impairment of neural conduction along the small and large axons of the visual pathways by time, corroborating the notion that NAION produces a post-retinal neural conduction delay for neurodegenerative mechanisms. [11] Our findings in NN eyes are in agreement with reports in NAION patients [69,70] describing permanent delay of optic nerve conduction persisting after acute injury.

By contrast, in NC eyes, we observed significant 60’ and 15’ VEP-IT shortening at the end of treatment and after wash-out periods (S2 Table). This suggests that OS-Citicoline may enhance post-retinal neural conduction along the small and large axons of the visual pathways.

It is worth noting that after OS-Citicoline administration, greater retinal and post-retinal functional improvement was observed in NC eyes with greater dysfunction at baseline (Fig 3B and 3D). This is consistent with our previous observation [39], in which glaucomatous patients were “better responders” to Citicoline eye drop treatment when greater impairment at baseline was present.

When we related VEP-IT with relative PERG-A, we did not detect any significant linear correlation (“S1A and S1B Fig”). This suggests that the amelioration of neural conduction along the visual pathways observed in NC eyes at the end of treatment was independent from improvement of retinal dysfunction. Therefore, these phenomena may be the result of two concomitant but unrelated factors: one at the retinal level (see above *“Retinal Ganglion Cells function*: *PERG data*”) and one at the post-retinal level.

Concerning the latter factor, our results can be explained based on the neuromodulatory effects of Citicoline as “dopaminergic-like activity”. [29,71] This is derived from a similar increase in visual acuity and neural conduction in amblyopic eyes using intramuscular or oral suspension Citicoline [72,73] or on the basis of results in Parkinson’s disease patients where Citicoline has been used as a complement to levodopa therapy. [74] Moreover, Citicoline may have potential neuromodulatory roles as demonstrated in conditions of cerebral hypoxia and ischemia, by increasing the levels and rate of synthesis of neurotransmitters such as acetylcholine, noradrenalin, and serotonin in certain brain areas [75].

In NC eyes, the differences between improvements in neural conduction were significantly linearly correlated with differences in the increase in VA and reduction of HFA-MD. Another property of Citicoline is to increase the level of consciousness [76], and this may also explain, at least in part, similar visual field changes observed after one vial (containing 500 mg of OS-Citicoline) per day treatment in glaucomatous patients. [53] However, in the present study, based on the linear correlation between improved neurofunctional (VEP-IT) and psychophysical (HFA-MD) parameters (“S1E and S1F Fig”), we can exclude attention level as exclusively producing visual field or visual acuity changes. This is consistent with the correlation between VEP and HFA-MD changes reported after 8 years of intramuscular treatment with Citicoline in glaucoma [37].

These findings collectively led us to the conclusion that OS-Citicoline has neuroenhancer effects on RGCs function and neural conduction along the visual pathways in NAION as in glaucoma [19,45] and other cerebrovascular and/or neurodegenerative disorders [77].

#### Neuroprotection

RNFL-T is the morphological index used in this study to describe the integrity of RGCs fibers forming the optic nerve. At baseline, compared to Controls, both NN and NC Groups presented significant reduction in RNFL-T in all sectors (Table 1), suggesting that a remarkable loss of nerve fibers characterizes the chronic stage of NAION. This is consistent with other previous reports, in which RNFL thinning was detected as early as one or two months from the acute onset of NAION [78–80].

After OS-Citicoline treatment and also after wash-out, RNFL-TO was either unmodified (in the majority of NC eyes: 84.21% and 78.95%, respectively) or improved (in a small percentage of NC eyes: 15.79% and 21.05%, respectively). No RNFL-TO worsening was detected (0% of NC eyes). In the NC Group, RNFL-TO was significantly increased with respect to baseline (“S3 Table”, Fig 4C).

These findings are relevant when compared with those observed in NN eyes, in which a significant progressive thinning of the overall RNFL was detected (Table 2). There is a paucity of literature on the progression of RNFL thinning in NAION, and the few reports showing RNFL stability during a follow up of 12–23 months are in disagreement with our findings, possible due to the different OCT apparatus used [81,82].

Since no other similar studies performed in humans have been reported in the literature, we are able to provide a possible explanation for the unmodified or improved RNFL thickness after treatment with OS-Citicoline exclusively on the basis of results obtained in experimental (cellular or animal) models [83–88]. Results from these studies highlight two notable properties of Citicoline: preventing RGCs death and fibers loss by controlling neuronal apoptosis and inducing regeneration of new-born RGCs neurites.

Effects counteracting neuronal degeneration evaluated in terms of reduced apoptosis and loss of synapses are derived from an *in vitro* study in rat retina cultures exposed to glutamate or to high glucose. Increasing concentrations of Citicoline infused in cultures improved glutamate uptake with consequent reduced neuronal membrane impairment and amelioration of synaptic connectivity [83]. Prevention of early loss of neuronal retinal cells by Citicoline was suggested by an *in vivo* mouse model of type 1 diabetes. In these mice, progressive retinal morphological impairment (thinning of RNFL and of RGCs complex) was detected by OCT assessment. The above-mentioned preventative effects of Citicoline (administered by eye drops) were hypothesized on the basis of reduced neuroretinal structural abnormalities. [84] Similarly, in the optic nerve crush model, with respect to PBS-treated eyes, rats treated with Citicoline combined with tauroursodeoxycholic acid and neurotrophin-4 showed a higher density of RCGs and higher number of RGCs axons [85].

Neuroregenerative effects of Citicoline were reported in a study performed in an animal model of cerebral ischemia. In rats injected daily with Citicoline, newly born neurons and migration of neural progenitors to the ischemic lesion and functional long-term somatosensitive recovery were observed [86].

Both the above-mentioned properties of Citicoline have been supported by a murine model of damaged RGCs cultures. After treatment with high concentration of Citicoline, rescue of RGCs (ascribed to anti-apoptotic effects in mitochondria-dependent cell death mechanism) and regeneration of neurites (well quantified by TUNEL positivity analysis) were observed. [87] Moreover, in cultured rat retinas exposed to high glucose and infused with Citicoline, brain-derived neurotrophic factor and neurotrophin-4, decreased number of apoptotic RGCs (due to suppression of caspase-9 and -3 activity), and increased number of regenerating neurites were observed compared to non-supplemented cultures [88].

These reports [83–88] led us to the conclusion that in human optic nerve neurodegeneration, Citicoline can stabilize (unmodified RNFL) or improve (increased RNFL) RGCs and their fibers structure. This may be considered a “neuroprotective” effect. Nevertheless, since our OCT method allowed us to evaluate optic nerve axons (i.e., RNFL), without selective evaluation of the morphological status of RGCs somata, we believe that it is more appropriate to define our morphological findings as “axoprotective effects”.

In NC eyes, the lack of a significant correlation between morphological (RNFL-T) and electrophysiological (PERG and VEP values) data suggests that OS-Citicoline has concomitant but unrelated functional and structural effects on RGCs axons.

## Conclusions

Our results, obtained in a human model of fast RGCs degeneration (NAION), suggest that OS-Citicoline treatment induces both neuroenhancer (improvement of RGCs function and neural conduction along visual pathways with related improvement of visual field defects) and neuroprotective (unmodified or improved RNFL morphological condition) effects.

A limitation of the present study was that the OCT apparatus only allowed us to evaluate optic nerve axons but not directly assess the morphological status of RGCs somata. It is likely that more newly derived OCT indexes (such as the ganglion cells-inner plexiform layer complex) [89,90] will help to further evaluate the neuroprotective effects of Citicoline. Nevertheless, our preliminary results need to be confirmed by further investigations on this topic, with a larger cohort of patients and longer observation time for both treatment and wash-out periods to observe the persistence of neuroenhancer and neuroprotective effects of OS-Citicoline over time.

## Supporting information

S1 Fig **Visual evoked potentials (VEP) implicit time individual differences between baseline and the end of Citicoline treatment (6 months minus baseline) in non-arteritic ischemic optic neuropathy (NAION) patients (NC Group) plotted as a function of the values of the corresponding differences of: (A, B)** Pattern electroretinogram (PERG) P50-N95 amplitude, **(C)** Retinal nerve fiber layer thickness (RNFL-T) Overall (compared with 60’ VEP), **(D)** RNFL-T Temporal (compared with 15’VEP), **(E, F)** Mean Deviation of Humphrey 24–2 perimetry (HFA). Pearson’s test was used for regression analysis and correlations.
(TIF)

S1 Table Changes in electrophysiological [Pattern Electroretinogram (PERG) P50-N95 Amplitudes, Visual Evoked Potentials (VEP) P100 Implicit Times and N75-P100 Amplitudes], morphological [Retinal nerve fiber layer thickness (RNFL) thickness] and psychophysical [Humphrey 24–2 perimetry (HFA) Mean Deviation (MD) and logMAR Visual Acuity (VA)] values after 6 and 9 months of follow-up in patients with non-arteritic ischemic optic neuropathy (NAION) treated with Citicoline in oral solution (NC Group, N = 19 eyes) and in untreated NAION patients (NN Group, N = 17 eyes). A, Amplitude; IT, implicit time; 60’ and 15’: visual stimuli in which each check subtended 60 and 15 minutes of the visual arc, respectively; TO, overall thickness; TS, superior thickness; TN, nasal thickness; TI, inferior thickness; TT, temporal thickness. Unmodified: within the 95% confidence test-retest limit. We considered as improved the values of PERG amplitude (A), RNFL thickness, MD, and VA with an increase with respect to baseline that exceeded the 95% confidence test-retest limit and values of VEP implicit time (IT) with a reduction with respect to baseline that exceeded the 95% confidence test-retest limit. We considered as worsened the values of PERG amplitude (A), RNFL thickness, MD, and VA with a reduction with respect to baseline that exceeded the 95% confidence test-retest limit and values of VEP implicit time (IT) with an increase with respect to baseline that exceeded the 95% confidence test-retest limit. N: number of eyes.
(DOCX)

S2 Table Mean of absolute values of Pattern Electroretinogram (PERG) P50-N95 Amplitudes, in Visual Evoked Potentials (VEP) P100 Implicit Times and N75-P100 Amplitudes observed in patients with non-arteritic ischemic optic neuropathy (NAION) treated with Citicoline in oral solution (NC Group, N = 19 eyes) and in untreated NAION patients (NN Group, N = 17 eyes) at baseline condition and after 6 and 9 months of follow-up. ANOVA: one-way analysis of variance. SD: 1 standard deviation; 60’ and 15’: visual stimuli in which each check subtended 60 and 15 minutes of the visual arc respectively; A, Amplitude; μV, microvolt; IT, Implicit Time; msec, milliseconds; N, number of eyes.
(DOCX)

S3 Table Mean of absolute values of retinal nerve fiber layer (RNFL) thickness observed in patients with non-arteritic ischemic optic neuropathy (NAION) treated with Citicoline in oral solution (NC Group, N = 19 eyes) and in untreated NAION patients (NN Group, N = 17 eyes) at baseline condition and after 6 and 9 months of follow-up. ANOVA: one-way analysis of variance. SD: 1 standard deviation; TO, overall thickness; TS, superior thickness; TN, nasal thickness; TI, inferior thickness; TT, temporal thickness; μ, micron; N, number of eyes.
(DOCX)

S1 File CONSORT 2010 checklist reporting information on the presented randomised trial. (DOC)
