# Correction: Neuroenhancement and neuroprotection by oral solution citicoline in non-arteritic ischemic optic neuropathy as a model of neurodegeneration: A randomized pilot study

**DOI:** 10.1371/journal.pone.0221313

**Published:** 2019-08-14

**Authors:** The PLOS ONE Staff

Due to a typesetting error, incorrect versions of Fig 3 and its corresponding caption are included in this article [1]. The publisher apologizes for the error. Please see the correct Fig 3 and caption here.

**Fig 3:**
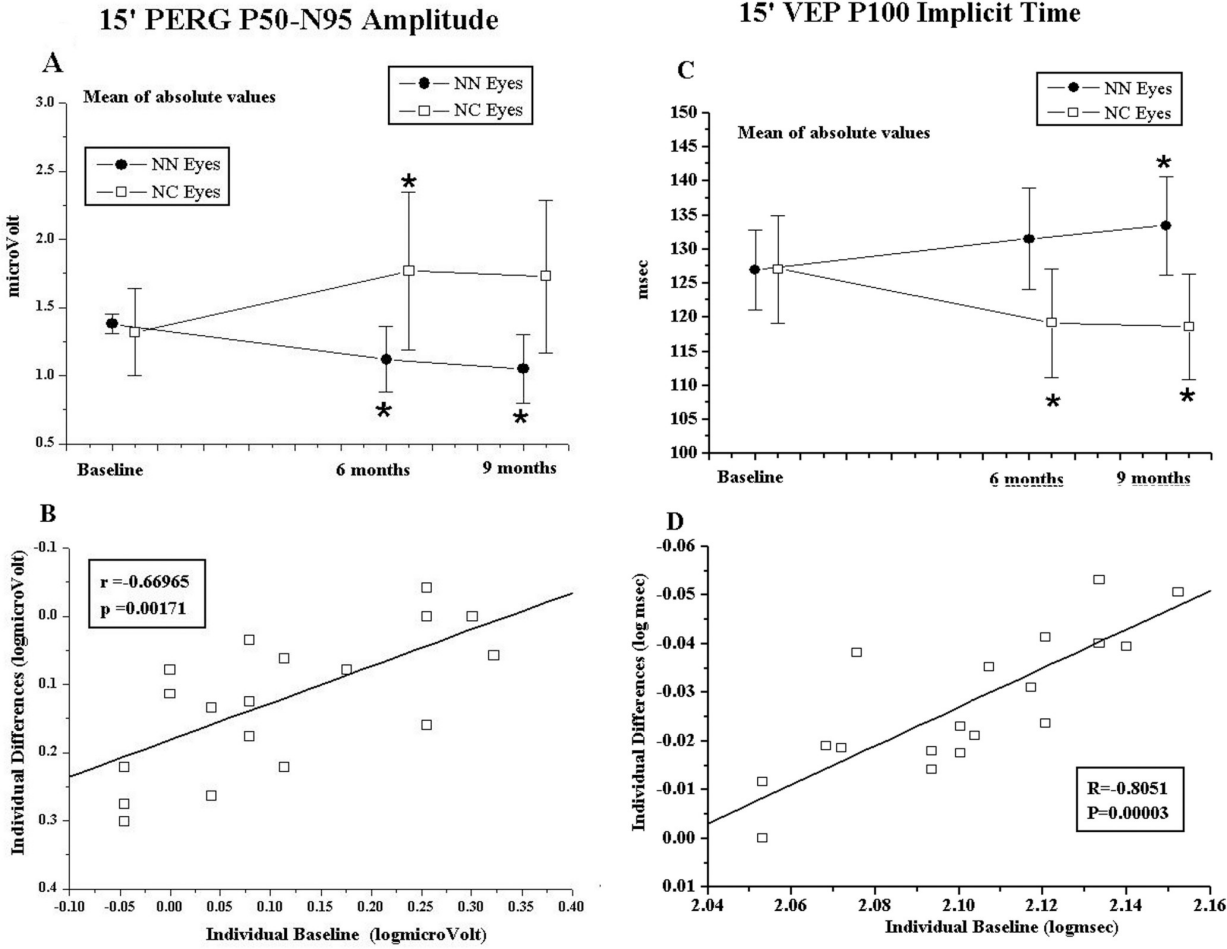
Pattern electroretinogram (PERG) and visual evoked potentials (VEP) P100 implicit time recorded in response to 15’ checks (15’) results. **(A)** Mean of absolute PERG P50-N95 Amplitude values observed in NC and NN Groups. * = ANOVA, p<0.01 in NN and NC Groups with respect to baseline. Vertical lines: one mean standard deviation. The statistical evaluation is reported in S2 Table. **(B)** Individual PERG P50-N95 Amplitude values observed in NC eyes at baseline plotted as a function of the values of the corresponding differences at the end of treatment (6 months minus baseline). Pearson’s test was used for regression analysis and linear correlation. **(C)** Mean of absolute VEP P100 implicit time values observed in NC and NN Groups. * = ANOVA, p<0.01 in NN and NC Groups with respect to baseline. Vertical lines: one mean standard deviation. Statistical evaluation is reported in “S2 Table”. **(D)** Individual VEP P100 implicit time values observed in NC eyes at baseline plotted as a function of the values of the corresponding differences at the end of treatment (6 months minus baseline). Pearson’s test was used for regression analysis and linear correlation.
