# Supplementary figures and images for: Neuroenhancement and neuroprotection by oral solution citicoline in non-arteritic ischemic optic neuropathy as a model of neurodegeneration: A randomized pilot study
Source: PLoS One. 2019 Jul 26;14(7):e0220435. doi: 10.1371/journal.pone.0220435 (PMC6660126; doi:10.1371/journal.pone.0220435)

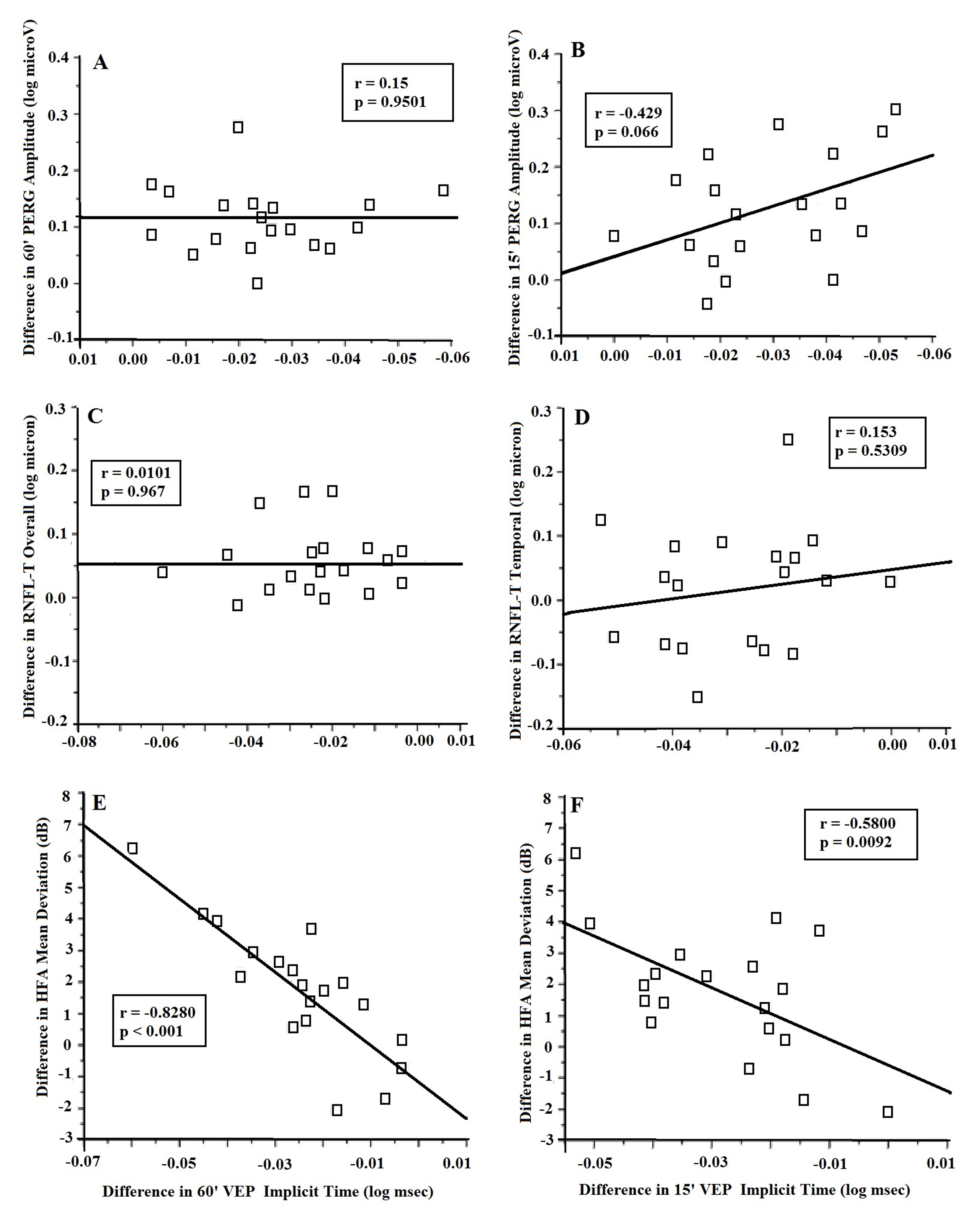

Supplement: S1 Fig — Visual evoked potentials (VEP) implicit time individual differences between baseline and the end of Citicoline treatment (6 months minus baseline) in non-arteritic ischemic optic neuropathy (NAION) patients (NC Group) plotted as a function of the values of the corresponding differences of: (A, B) Pattern electroretinogram (PERG) P50-N95 amplitude, (C) Retinal nerve fiber layer thickness (RNFL-T) Overall (compared with 60’ VEP), (D) RNFL-T Temporal (compared with 15’VEP), (E, F) Mean Deviation of Humphrey 24–2 perimetry (HFA). Pearson’s test was used for regression analysis and correlations. (TIF) [file pone.0220435.s001.tif]
