# Supplementary material for: Neuroenhancement and neuroprotection by oral solution citicoline in non-arteritic ischemic optic neuropathy as a model of neurodegeneration: A randomized pilot study
Source: PLoS One. 2019 Jul 26;14(7):e0220435. doi: 10.1371/journal.pone.0220435 (PMC6660126; doi:10.1371/journal.pone.0220435)
Supplement: S1 Table — A, Amplitude; IT, implicit time; 60’ and 15’: visual stimuli in which each check subtended 60 and 15 minutes of the visual arc, respectively; TO, overall thickness; TS, superior thickness; TN, nasal thickness; TI, inferior thickness; TT, temporal thickness. Unmodified: within the 95% confidence test-retest limit. We considered as improved the values of PERG amplitude (A), RNFL thickness, MD, and VA with an increase with respect to baseline that exceeded the 95% confidence test-retest limit and values of VEP implicit time (IT) with a reduction with respect to baseline that exceeded the 95% confidence test-retest limit. We considered as worsened the values of PERG amplitude (A), RNFL thickness, MD, and VA with a reduction with respect to baseline that exceeded the 95% confidence test-retest limit and values of VEP implicit time (IT) with an increase with respect to baseline that exceeded the 95% confidence test-retest limit. N: number of eyes. (DOCX) [file pone.0220435.s002.docx]

**S1 Table.**

| **6 months minus baseline difference** | | | | | | | | | | | | |
| --- | --- | --- | --- | --- | --- | --- | --- | --- | --- | --- | --- | --- |
|  | **Group NN eyes (N=17)** | | | | | | **Group NC eyes (N=19)** | | | | | |
|  | **unmodified** | | **Improvement** | | **worsening** | | **unmodified** | | **Improvement** | | **worsening** | |
|  | **N** | **%** | **­N** | **%** | **N** | **%** | **N** | **%** | **­N** | **%** | **N** | **%** |
| **60’ PERG P50-N95 A** | 12 | 70.59 | 1 | 5.882 | 4 | 23.53 | 5 | 26.32 | 14 | 73.68 | 0 | 0 |
| **60’ VEP P100 IT** | 9 | 52.94 | 0 | 0 | 8 | 47.06 | 6 | 31.58 | 13 | 68.42 | 0 | 0 |
| **60’ VEP N75-P100 A** | 14 | 82.35 | 0 | 0 | 3 | 17.65 | 6 | 31.58 | 13 | 68.42 | 0 | 0 |
| **15’ PERG P50-N95 A** | 12 | 70.59 | 0 | 0 | 5 | 29.41 | 6 | 31.58 | 13 | 68.42 | 0 | 0 |
| **15’ VEP P100 IT** | 13 | 76.47 | 0 | 0 | 4 | 23.53 | 5 | 26.32 | 14 | 73.68 | 0 | 0 |
| **15’ VEP N75-P100 A** | 13 | 76.47 | 0 | 0 | 4 | 23.53 | 12 | 63.16 | 7 | 36.84 | 0 | 0 |
| **RNFL-TO** | 10 | 58.82 | 0 | 0 | 7 | 41.18 | 16 | 84.21 | 3 | 15.79 | 0 | 0 |
| **RNFL-TS** | 11 | 64.71 | 0 | 0 | 6 | 35.29 | 9 | 47.37 | 9 | 47.37 | 1 | 5.26 |
| **RNFL-TN** | 11 | 64.71 | 0 | 0 | 6 | 35.29 | 15 | 78.95 | 4 | 21.05 | 0 | 0 |
| **RNFL-TI** | 12 | 70.59 | 0 | 0 | 5 | 29.41 | 11 | 57.89 | 7 | 36.84 | 1 | 5.26 |
| **RNFL-TT** | 8 | 47.06 | 0 | 0 | 9 | 52.94 | 8 | 42.11 | 5 | 26.32 | 6 | 31.6 |
| **HFA MD** | 5 | 29.41 | 0 | 0 | 12 | 70.59 | 3 | 15.79 | 14 | 73.68 | 2 | 10.5 |
| **VA** | 12 | 70.59 | 0 | 0 | 5 | 29.41 | 11 | 57.89 | 7 | 36.84 | 1 | 5.27 |
| **9 months minus baseline difference** | | | | | | | | | | | | |
|  | **Group NN eyes (N=17)** | | | | | | **Group NC eyes (N=10)** | | | | | |
|  | **unmodified** | | **improvement** | | **worsening** | | **unmodified** | | **improvement** | | **worsening** | |
|  | **N** | **%** | **­N** | **%** | **N** | **%** | **N** | **%** | **­N** | **%** | **N** | **%** |
| **60’ PERG P50-N95 A** | 9 | 52.94 | 0 | 0 | 8 | 47.06 | 8 | 47.06 | 11 | 64.71 | 0 | 0 |
| **60’ VEP P100 IT** | 6 | 35.29 | 0 | 0 | 11 | 64.71 | 6 | 35.29 | 13 | 76.47 | 0 | 0 |
| **60’ VEP N75-P100 A** | 10 | 58.82 | 0 | 0 | 7 | 41.18 | 13 | 76.47 | 5 | 29.41 | 1 | 5.88 |
| **15’’ PERG P50-N95 A** | 10 | 58.82 | 0 | 0 | 7 | 41.18 | 10 | 58.82 | 9 | 52.94 | 0 | 0 |
| **15’ VEP P100 IT** | 9 | 52.94 | 0 | 0 | 8 | 47.06 | 8 | 47.06 | 11 | 64.71 | 0 | 0 |
| **15’ VEP N75-P100 A** | 5 | 29.41 | 0 | 0 | 12 | 70.59 | 8 | 47.06 | 10 | 58.82 | 1 | 5.88 |
| **RNFL-TO** | 7 | 41.18 | 0 | 0 | 10 | 58.82 | 15 | 88.24 | 4 | 23.53 | 0 | 0 |
| **RNFL-TS** | 6 | 35.29 | 1 | 5.88 | 10 | 58.82 | 11 | 64.71 | 8 | 47.06 | 0 | 0 |
| **RNFL-TN** | 7 | 41.18 | 0 | 0 | 10 | 58.82 | 13 | 76.47 | 6 | 35.29 | 0 | 0 |
| **RNFL-TI** | 12 | 70.59 | 0 | 0 | 5 | 29.41 | 14 | 82.35 | 5 | 29.41 | 0 | 0 |
| **RNFL-TT** | 4 | 23.53 | 2 | 11.8 | 11 | 64.71 | 7 | 41.18 | 6 | 35.29 | 6 | 35.29 |
| **HFA MD** | 4 | 23.53 | 0 | 0 | 13 | 76.47 | 5 | 29.41 | 12 | 70.59 | 2 | 11.76 |
| **VA** | 9 | 52.94 | 1 | 5.88 | 7 | 41.18 | 10 | 52.63 | 8 | 42.11 | 1 | 5.26 |
