# Supplementary material for: Neuroenhancement and neuroprotection by oral solution citicoline in non-arteritic ischemic optic neuropathy as a model of neurodegeneration: A randomized pilot study
Source: PLoS One. 2019 Jul 26;14(7):e0220435. doi: 10.1371/journal.pone.0220435 (PMC6660126; doi:10.1371/journal.pone.0220435)
Supplement: S2 Table — ANOVA: one-way analysis of variance. SD: 1 standard deviation; 60’ and 15’: visual stimuli in which each check subtended 60 and 15 minutes of the visual arc respectively; A, Amplitude; μV, microvolt; IT, Implicit Time; msec, milliseconds; N, number of eyes. (DOCX) [file pone.0220435.s003.docx]

**S2 Table.**

|  | **Group NN (N=17)** | | | | **Group NC (N=19)** | | | |
| --- | --- | --- | --- | --- | --- | --- | --- | --- |
|  |  |  | Anova vs Baseline | |  |  | Anova vs Baseline | |
|  | **Mean** | **SD** |  |  | **Mean** | **SD** |  |  |
|  |  |  | f(1,33)= | P= |  |  | f(1,37) | P= |
| **60’ PERG P50-N95 A (µV)** | | | | | | | | |
| **Baseline** | 1.52 | 0.23 |  |  | 1.34 | 0.36 |  |  |
| **6 months** | 1.27 | 0.25 | 9.23 | 0.005 | 1.75 | 0.55 | 7.47 | 0.009 |
| **9 months** | 1.16 | 0.25 | 19.11 | 0.0001 | 1.80 | 0.56 | 9.12 | 0.005 |
| **60’VEP P100 IT (msec)** | | | | | | | | |
| **Baseline** | 126.71 | 5.87 |  |  | 127.52 | 7.86 |  |  |
| **6 months** | 132.32 | 7.51 | 5.84 | 0.022 | 120.37 | 7.98 | 7.52 | 0.009 |
| **9 months** | 134.11 | 7.17 | 10.92 | 0.002 | 118.51 | 7.68 | 12.42 | 0.001 |
| **60’ VEP N75-P100 A (µV)** | | | | | | | | |
| **Baseline** | 5.36 | 2.84 |  |  | 4.02 | 2.66 |  |  |
| **6 months** | 4.12 | 2.01 | 2.14 | 0.154 | 6.12 | 2.68 | 5.86 | 0.021 |
| **9 months** | 3.46 | 1.64 | 5.66 | 0.023 | 6.33 | 3.15 | 5.99 | 0.019 |
| **15’ PERG P50-N95 A (µV)** | | | | | | | | |
| **Baseline** | 1.38 | 0.28 |  |  | 1.32 | 0.39 |  |  |
| **6 months** | 1.12 | 0.25 | 7.56 | 0.009 | 1.77 | 0.58 | 7.92 | 0.008 |
| **9 months** | 1.05 | 0.26 | 12.4 | 0.001 | 1.73 | 0.57 | 6.69 | 0.014 |
| **15’VEP P100 IT (msec)** | | | | | | | | |
| **Baseline** | 126.88 | 6.50 |  |  | 127.05 | 8.35 |  |  |
| **6 months** | 131.52 | 6.72 | 4.20 | 0.049 | 119.12 | 8.69 | 8.27 | 0.007 |
| **9 months** | 133.41 | 6.08 | 9.13 | 0.005 | 118.62 | 8.75 | 9.20 | 0.004 |
| **15’ VEP N75-P100 A (µV)** | | | | | | | | |
| **Baseline** | 5.87 | 2.53 |  |  | 4.64 | 2.64 |  |  |
| **6 months** | 4.66 | 1.73 | 2.63 | 0.115 | 5.77 | 2.86 | 1.59 | 0.215 |
| **9 months** | 3.37 | 1.52 | 12.21 | 0.001 | 6.19 | 2.85 | 3.03 | 0.090 |
