# Supplementary material for: Neuroenhancement and neuroprotection by oral solution citicoline in non-arteritic ischemic optic neuropathy as a model of neurodegeneration: A randomized pilot study
Source: PLoS One. 2019 Jul 26;14(7):e0220435. doi: 10.1371/journal.pone.0220435 (PMC6660126; doi:10.1371/journal.pone.0220435)
Supplement: S3 Table — ANOVA: one-way analysis of variance. SD: 1 standard deviation; TO, overall thickness; TS, superior thickness; TN, nasal thickness; TI, inferior thickness; TT, temporal thickness; μ, micron; N, number of eyes. (DOCX) [file pone.0220435.s004.docx]

**S3 Table.**

|  | **Group NN (N=17)** | | | | **Group NC (N=19)** | | | |
| --- | --- | --- | --- | --- | --- | --- | --- | --- |
|  |  |  | **ANOVA vs Baseline** | |  |  | **ANOVA vs Baseline** | |
|  | **Mean** | **SD** |  |  | **Mean** | **SD** |  |  |
|  |  |  | f(1,33)= | P= |  |  | f(1,37) | P= |
| **RNFL-TO (µ)** | | | | | | | | |
| **Baseline** | 63.99 | 8.44 |  |  | 57.71 | 9.43 |  |  |
| **6 months** | 57.02 | 8.08 | 7.46 | 0.010 | 65.08 | 6.92 | 7.54 | 0.009 |
| **9 months** | 52.45 | 9.41 | 13.02 | 0.001 | 65.73 | 7.40 | 8.51 | 0.006 |
| **RNFL-TS (µ)** | | | | | | | | |
| **Baseline** | 69.91 | 9.25 |  |  | 61.89 | 14.02 |  |  |
| **6 months** | 62.98 | 12.33 | 3.44 | 0.073 | 74.41 | 11.40 | 9.12 | 0.005 |
| **9 months** | 57.88 | 13.36 | 9.32 | 0.004 | 74.87 | 8.66 | 11.79 | 0.001 |
| **RNFL-TN (µ)** | | | | | | | | |
| **Baseline** | 54.59 | 13.91 |  |  | 46.26 | 9.29 |  |  |
| **6 months** | 48.90 | 11.46 | 1.69 | 0.203 | 50.76 | 10.03 | 2.06 | 0.160 |
| **9 months** | 45.29 | 9.19 | 5.29 | 0.028 | 52.73 | 8.92 | 4.80 | 0.035 |
| **RNFL-TI (µ)** | | | | | | | | |
| **Baseline** | 76.95 | 16.72 |  |  | 75.84 | 21.34 |  |  |
| **6 months** | 69.03 | 14.11 | 2.23 | 0.145 | 86.45 | 14.26 | 3.25 | 0.080 |
| **9 months** | 65.35 | 15.82 | 4.32 | 0.045 | 88.01 | 20.04 | 3.28 | 0.078 |
| **RNFL-TT (µ)** | | | | | | | | |
| **Baseline** | 54.53 | 16.17 |  |  | 46.84 | 10.32 |  |  |
| **6 months** | 41.47 | 10.71 | 7.69 | 0.009 | 48.69 | 12.77 | 0.24 | 0.625 |
| **9 months** | 41.29 | 11.27 | 7.66 | 0.009 | 47.33 | 9.31 | 0.02 | 0.878 |
