# Supplementary material for: Neuroenhancement and neuroprotection by oral solution citicoline in non-arteritic ischemic optic neuropathy as a model of neurodegeneration: A randomized pilot study
Source: PLoS One. 2019 Jul 26;14(7):e0220435. doi: 10.1371/journal.pone.0220435 (PMC6660126; doi:10.1371/journal.pone.0220435)
Supplement: S1 File — (DOC) [file pone.0220435.s005.doc]

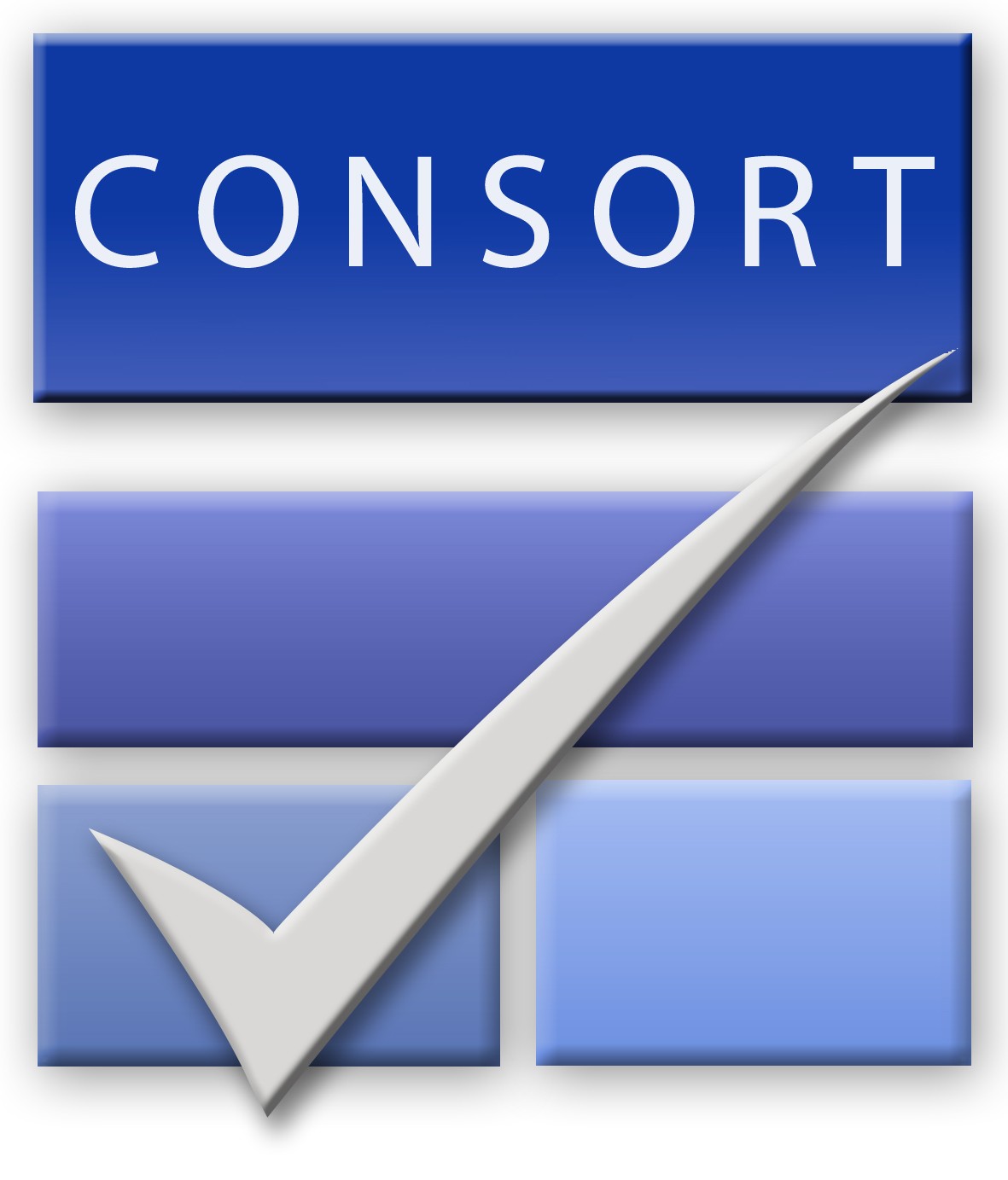
CONSORT 2010 checklist of information to include when reporting a randomised trial*

| Section/Topic | Item No | Checklist item | | Reported on page No |
| --- | --- | --- | --- | --- |
| Title and abstract | | | | |
|  | 1a | Identification as a randomised trial in the title | p.1-line 2 | |
|  | 1b | Structured summary of trial design, methods, results, and conclusions (for specific guidance see CONSORT for abstracts) | p.2- line 27-51 | |
| Introduction | | | | |
| Background and objectives | 2a | Scientific background and explanation of rationale | | p.3-4 line 54-106 |
|  | 2b | Specific objectives or hypotheses | | p.4-5 line 107-134 |
| Methods | | | | |
| Trial design | 3a | Description of trial design (such as parallel, factorial) including allocation ratio | | p.6-7 line 172-207 |
|  | 3b | Important changes to methods after trial commencement (such as eligibility criteria), with reasons | | N/A |
| Participants | 4a | Eligibility criteria for participants | | p.5-6 line 145-161 |
|  | 4b | Settings and locations where the data were collected | | p.6 line 179-180 |
| Interventions | 5 | The interventions for each group with sufficient details to allow replication, including how and when they were actually administered | | p.6-7 line 181-207 |
| Outcomes | 6a | Completely defined pre-specified primary and secondary outcome measures, including how and when they were assessed | | p.6-8 line 181-257 |
|  | 6b | Any changes to trial outcomes after the trial commenced, with reasons | | p.6 line 172-175 |
| Sample size | 7a | How sample size was determined | | p.9 line 256-266 |
|  | 7b | When applicable, explanation of any interim analyses and stopping guidelines | | N/A |
| Randomisation: |  |  | |  |
| Sequence generation | 8a | Method used to generate the random allocation sequence | | p.6 line 181-186 |
|  | 8b | Type of randomisation; details of any restriction (such as blocking and block size) | | p.6 line 181-186 |
| Allocation concealment mechanism | 9 | Mechanism used to implement the random allocation sequence (such as sequentially numbered containers), describing any steps taken to conceal the sequence until interventions were assigned | | p.6 line 181-186 |
| Implementation | 10 | Who generated the random allocation sequence, who enrolled participants, and who assigned participants to interventions | | p.6 line 181-186 |
| Blinding | 11a | If done, who was blinded after assignment to interventions (for example, participants, care providers, those assessing outcomes) and how | | p.6 line 176-177; p.7 line 200-203 |
|  | 11b | If relevant, description of the similarity of interventions | | p.4 103-106 |
| Statistical methods | 12a | Statistical methods used to compare groups for primary and secondary outcomes | | p.9 line 256-286 |
|  | 12b | Methods for additional analyses, such as subgroup analyses and adjusted analyses | | N/A |
| Results | | | | |
| Participant flow (a diagram is strongly recommended) | 13a | For each group, the numbers of participants who were randomly assigned, received intended treatment, and were analysed for the primary outcome | | p.6-7 line 189-194 |
|  | 13b | For each group, losses and exclusions after randomisation, together with reasons | | p.6-7 line 189-194, p.7 line 206-207 |
| Recruitment | 14a | Dates defining the periods of recruitment and follow-up | | p.6-7 line 179-207 |
|  | 14b | Why the trial ended or was stopped | | N/A |
| Baseline data | 15 | A table showing baseline demographic and clinical characteristics for each group | | p.10-11 line 323-324 |
| Numbers analysed | 16 | For each group, number of participants (denominator) included in each analysis and whether the analysis was by original assigned groups | | p.10-18 line 289-529 |
| Outcomes and estimation | 17a | For each primary and secondary outcome, results for each group, and the estimated effect size and its precision (such as 95% confidence interval) | | p.10-18 line 289-529 |
|  | 17b | For binary outcomes, presentation of both absolute and relative effect sizes is recommended | | N/A |
| Ancillary analyses | 18 | Results of any other analyses performed, including subgroup analyses and adjusted analyses, distinguishing pre-specified from exploratory | | N/A |
| Harms | 19 | All important harms or unintended effects in each group (for specific guidance see CONSORT for harms) | | p.18 line 528-529 |
| Discussion | | | | |
| Limitations | 20 | Trial limitations, addressing sources of potential bias, imprecision, and, if relevant, multiplicity of analyses | | p.22 line 667-672 |
| Generalisability | 21 | Generalisability (external validity, applicability) of the trial findings | | p.22 line 662-676 |
| Interpretation | 22 | Interpretation consistent with results, balancing benefits and harms, and considering other relevant evidence | | p.18-21 line 532-659 |
| Other information | | | |  |
| Registration | 23 | Registration number and name of trial registry | | p.6 line 175 |
| Protocol | 24 | Where the full trial protocol can be accessed, if available | | p.22 line 681-682 |
| Funding | 25 | Sources of funding and other support (such as supply of drugs), role of funders | | p.22 line 680-681 |

*We strongly recommend reading this statement in conjunction with the CONSORT 2010 Explanation and Elaboration for important clarifications on all the items. If relevant, we also recommend reading CONSORT extensions for cluster randomised trials, non-inferiority and equivalence trials, non-pharmacological treatments, herbal interventions, and pragmatic trials. Additional extensions are forthcoming: for those and for up to date references relevant to this checklist, see [www.consort-statement.org](http://www.consort-statement.org/).
